# Multibody cofactor and substrate molecular recognition in the *myo*-inositol monophosphatase enzyme

**DOI:** 10.1038/srep30275

**Published:** 2016-07-21

**Authors:** Noelia Ferruz, Gary Tresadern, Antonio Pineda-Lucena, Gianni De Fabritiis

**Affiliations:** 1 Computational Biophysics Laboratory (GRIB-IMIM), Universitat Pompeu Fabra, Barcelona Biomedical Research Park (PRBB), Doctor Aiguader 88, 08003, Barcelona, Spain; 2 Acellera, Barcelona Biomedical Research Park, C Dr Aiguader 88, 08003, Barcelona, Spain; 3 Research Informatics, Janssen Research and Development, Janssen Cilag S A, Calle Jarama 75, Poligono Industrial, Toledo 45007, Spain; 4 Centro de Investigación Príncipe Felipe, 46012 Valencia, Spain; 5 Institució Catalana de Recerca i Estudis Avançats (ICREA), Passeig Lluis Companys 23, 08010 Barcelona, Spain

## Abstract

Molecular recognition is rarely a two-body protein-ligand problem, as it often involves the dynamic interplay of multiple molecules that together control the binding process. Myo-inositol monophosphatase (IMPase), a drug target for bipolar disorder, depends on 3 Mg^2+^ ions as cofactor for its catalytic activity. Although the crystallographic pose of the pre-catalytic complex is well characterized, the binding process by which substrate, cofactor and protein cooperate is essentially unknown. Here, we have characterized cofactor and substrate cooperative binding by means of large-scale molecular dynamics. Our study showed the first and second Mg^2+^ ions identify the binding pocket with fast kinetics whereas the third ion presents a much higher energy barrier. Substrate binding can occur in cooperation with cofactor, or alone to a binary or ternary cofactor-IMPase complex, although the last scenario occurs several orders of magnitude faster. Our atomic description of the three-body mechanism offers a particularly challenging example of pathway reconstruction, and may prove particularly useful in realistic contexts where water, ions, cofactors or other entities cooperate and modulate the binding process.

Bipolar disorder is a serious medical illness where episodes of mania alternate with depression. It currently affects more than 254 million people worldwide and is one of the major causes of loss of health and suicide in the middle-aged population^1^. Since the anti-manic properties of lithium were first reported more than 60 years ago^2^, it has been the most widely used treatment for bipolar disorder. Unfortunately, the ion’s therapeutic window is very narrow and it is accompanied by severe toxicity issues and side-effects such as tremors, frequent urination, thyroid problems, weight gain and kidney failure^3^. Therefore, it is desirable to replace it with a more harmless treatment.

The discovery that lithium intake diminishes brain inositol levels^4^ led to the formulation of the ‘Inositol depletion hypothesis^4^’ where the ion is proposed to mitigate neurotransmitters in the phosphatidyl inositol (PI) pathway (Fig. 1), overactive in bipolar patients^5^. Myo-inositol monophosphatase (IMPase) plays a key role in the PI pathway, by hydrolyzing *myo*-inositol monophosphate (IP). IMPase’s activity in patients suffering from bipolar disorder is assumed to be higher than normal, thus increasing myo-inositol levels. IMPase is also specifically inhibited by therapeutic (0.5–1.5 mM) concentrations of lithium^6^, and that is why it has been traditionally proposed as the putative target of the inositol depletion theory^7^,^8^. Accordingly, IMPase has been the subject of major industrial and academic research for bipolar treatment, and despite the myriad of inhibitors that have been tested over the last years, all have shown poor bioavailability or difficulties to reach the site of action *in vivo*^8^,^9^,^10^,^11^,^12^.

There are two main reasons for the failure in finding a bioavailable drug inhibiting IMPase. Firstly, the structure of IMPase reveals a difficult binding pocket for drug-like compounds. More concretely, mammalian IMPase have been crystalized from murine^13^, bovine^14^ and human^15^ brain and show a homodimer of 60 kDa, with each subunit consisting of a penta-layered αβαβα sandwich formed by alternating 9 α-helices and 13 β-strands (Fig. 2a). The active site of IMPase is a highly hydrophilic cavity lying beneath a β-hairpin region which is thought to play a critical role in the enzyme function^16^,^17^,^18^. To recognize the IP substrate the catalytic cavity is a highly polar pocket which favors polar charged compounds, typically unable to cross the blood-brain barrier (BBB)^19^. Secondly, although the structural conformation upon substrate and cofactor binding is well defined, its kinetic mechanism is still not clear. A recently solved human crystal IMPase structure in complex with Mg^2+^ and phosphate showed a catalytic pocket with 3 Mg^2+^ and superimposable with previous structures^13^. Mg^2+^ in site I, to which we will refer as Mg-I throughout this work, binds Glu70, Asp90 the carboxyl group of Ile92, three water molecules and the phosphate group. Mg^2+^ in site II (Mg-II) is coordinated with Asp90, Asp93, Asp220 the phosphate group and three water molecules, one being shared with Mg-I. The more external Mg^2+^ site III (Mg-III) is only coordinated by Glu70, the phosphate group and 5 water molecules (Fig. 2b). Different experiments have suggested that the three Mg^2+^ must occupy the catalytic pocket for the accomplishment of the reaction^14^,^20^,^21^. Attempts to quantify Mg^2+^ binding showed that the three ions bind with decreasing affinity: Mg-I with a K_D_ of 300 μM^22^, Mg-II, K_D_ = 3.9 mM^23^ and low affinity Mg-III. Mg^2+^ concentration in neurons range from 0.5 to 1 mM and therefore the real occupancy at physiological conditions is unclear^24^. Whereas some studies proved the enzyme is doubly bound in neurons and the third Mg^2+^ binds after substrate^17^, another suggested the presence of three Mg^2+^ in the absence of substrates^14^.

It is therefore important to determine the mechanism of binding and the most populated states of the protein under physiological conditions in order to provide the basis for the rational design of new inhibitors. Here, we have performed an unprecedented 0.8 milliseconds of all-atom high-throughput molecular dynamics simulations in order to ascertain the concrete mechanism of binding of Mg^2+^ and the pathway of binding of the natural substrate.

## Results

In all *in-silico* binding analyses, full kinetic and thermodynamic data were obtained by performing free-ligand binding^24^, all-atom molecular dynamics simulations with the ACEMD^25^ molecular dynamics software on the distributed computing project GPUGRID^26^. The data were analysed using the HTMD software^27^ (available at http://www.htmd.org) and a Markov state modelling (MSM)^28^ method able to produce quantitative estimations of k_on_, k_off_ and ∆G^0^. MSM analyses have been successfully used in a wide range of problems from ligand binding^24^,^29^ to the characterisation of protein folding^28^ and intrinsically disordered protein dynamics^30^. In this work, we provide a comprehensive study on IMPase enzyme. By means of HTMD, we explain the full picture of cofactor and substrate binding. A total of 6 simulation batches have been performed (Table 1). Four of them focused on understanding the IMPase mode of action in the absence of substrates or inorganic molecules, and the other two, on the natural substrate (IP)’s kinetic mechanism. More details on the specific simulation parameters are provided in the Methods section.

### IMPase mode of action in the absence of substrates

**Figure 3:**
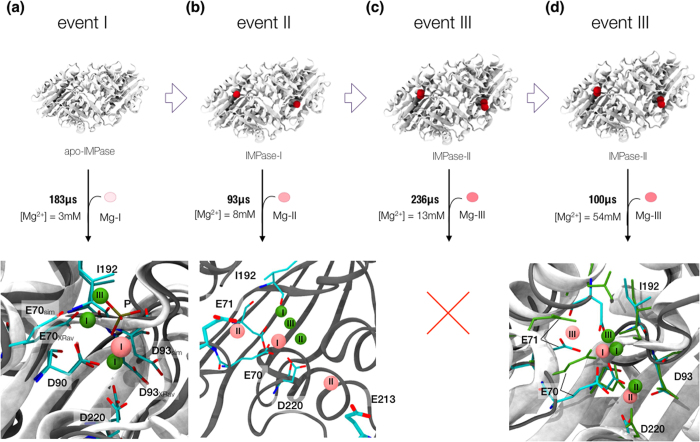
Summary of results for Mg^2+^ binding. Four *in-silico* experiments were performed to examine the ion’s binding events. **(a)** Binding of Mg-I to IMPase. One Mg^2+^ ion was placed around apo-IMPase. The ion bound the pocket with fast kinetics coordinating both I and II site’s residues. Superimposed with the crystal pose, the figure shows Mg-I’s binding mode in pink and the crystallographic ions in green. **(b)** Binding of Mg-II to IMPase, with Mg-I coordinating sites I and II as obtained in (**a**). The ion identified the pocket in two distinct poses in this timescale (pink), not corresponding to the crystallographic poses (green, see text). **(c)** Binding of Mg-III to IMPase at low concentration. 236 μs of simulation time did not show any binding event. **(d)** Binding of Mg-III to IMPase at high concentration. One binding event per subunit was registered, computing an on-rate of **~**10^5^ s^−1^ M^−1^. While Mg- I and II recapitulate the expected binding mode (residues in the simulation are shown in cyan, while X-ray residues and ions are shown in green), Mg-III’s binds more loosely interacting with E71.

Crystallographic studies have shown that there are three Mg^2+^ ions per subunit in the presence of substrates or inorganic phosphate^13^,^14^,^15^,^31^. However, some studies have proved IMPase is doubly bound in neurons and the third Mg^2+^ binds after the substrate^17^. Given the mild affinities of Mg^2+^ in site-I and II, and the low Mg^2+^ concentration in neurons (0.5–1 mM) is unclear what is the occupancy of IMPase prior to substrate binding. Here, we have examined the binding of the three Mg^2+^ ions by looking at the recognition process one ion at a time (Fig. 3). For the sake of simplicity, we will call binding event of Mg-I as event I, and subsequently event II and event III throughout the study.

### Binding of Mg-I: event I

For the analysis of event I, one single Mg^2+^ ion was placed free in solution around the *apo*-IMPase dimer giving a concentration of 3 mM, at which IMPase is maximally active^32^. The ion was placed at least 15 Å distance away from the protein in the initial systems’ coordinates, such as it could spontaneously identify its binding pocket without any bias. Using an adaptive sampling scheme^33^, more than 4500 trajectories of 40 ns were performed in order to compute the binding affinity and rate constants against each of the monomers. We analyzed the event I by performing one independent MSM analysis per subunit (see Methods), which provided remarkably consistent kinetic and affinity estimations between the monomers (Table 2). In quantitative terms, the standard free energy of binding is computed to be −3.8 ± 0.1 kcal/mol, slightly higher than previous dialysis (−4.8 kcal/mol)^22^ and fluorescence (−4.6 kcal/mol)^34^ experiments. There are no available crystal structures containing only one Mg^2+^ bound, however, in binary or ternary complexes Mg-I appears coordinated to three protein residues (Glu70, Asp90, Ile92) and three water molecules. Here, in the absence of Mg-II we observed that it tends to coordinate both characteristic site I and II residues and one single water molecule (Fig. 3). The transition to this pose occurs with very fast kinetics, in the order of 10^8^ M^−1^ s^−1^.

### Binding of Mg-II: event II

Reconstruction of event II was performed in a similar fashion. Mg-I was placed on each monomer coordinating both site I and II residues as obtained in event I’s analysis. Then a single Mg-II ion was placed free in solution, at least 15 Å distance from the protein. Accounting for three total Mg^2+^ ions, the concentration was 8 mM. We then performed another MSM analysis per monomer, as previously done for event I’s analysis. The analysis provided two poses with similar affinity in the first monomer, whereas in the other we lacked statistics to get a converged model. Looking at the first monomer’s poses, it was observed Mg-II tended to approach the catalytic pocket by interacting in two different binding sites other than the crystallographic one, as was site-II in coordination with Mg-I. Both poses are in the vicinity of Mg-I, the first interacting with Glu213, and the other with Glu71 (Fig. 3b, event II). We then analyzed Mg-I’s stability, in order to see transitions towards the crystallographic site. Taking into account the 277 μs produced in this and the previous set, we performed a root mean squared deviation (RMSD) of Mg-I aligning all Cα atoms in the protein against the crystal coordinates in 4AS4^13^. Only in 4 trajectories out of more than 5000, Mg-I evolved to the crystallographic position with an RMSD lower than 1 Å. Thorne *et al*. performed stopped-flow fluorescence spectroscopy studies to determine association and dissociation constants for Mg-I and II^34^. The study showed a slow increase in fluorescence after a rapid binding of Mg-I, suggesting that Mg-I binding is followed by a subtle structural rearrangement in the microenvironment of site I. The obtained on-rate was 4.4 ± 0.18·10^5^ M^−1^ s^−1^, whereas our estimates for Mg-I’s kinetics revealed a much faster process. Hence, event I showed very fast kinetics in our experiment, and our analysis did not recover the crystal pose, contrary to the equilibrium-based fluorescence experiments. Although our pose and the crystallographic one are only 3.3 Å RMSD away, subtle differences in neighboring atoms confer completely different octahedral coordination for Mg-I. We argue that the rearrangement of the negatively charged residues around these ions needed to reach its exact coordination found in X-ray is plausibly a much slower process in line with the experimental observations. Note that given the experimental kinetics, in order to sample such slow rearrangements, it would be needed to produce multi-millisecond simulations length that is beyond current capabilities.

### Binding of Mg-III: event III

For the analysis of event III, 236 μs of simulation time were produced. Mg-III was placed around IMPase, whereas Mg-I and II were located in their X-ray sites in both subunits this time, as the rearrangement to crystallographic positions was shown to be a very slow process. Interestingly, performing an RMSD analysis of the 3900 trajectories against the coordinates in 4AS4^13^ did not provide any spontaneous event III under these low concentration conditions. By performing an MSM analysis, we observed that at this concentration and timescale Mg-III binds at the interface between monomers (Fig. S1). Anticipating that event III is governed by very slow kinetics, we increased the concentration to 54 mM, with a total of 20 Mg^2+^ ions in the box. In 100 μs of simulation time, only 1 complete binding event was recovered per monomer, providing an RMSD of 2 Å to the crystal position of Mg-III^13^. The computed binding frequency is of the order of 0.01 μs^−1^, which, taking into account the concentration yields an on-rate of 1.85·10^5^ M^−1^ s^−1^.

Mg^2+^ is known to exert a bimodal activation on IMPase depending on its concentration. At low concentrations, as in neurons, Mg^2+^ acts as an activator being maximally active at 1 mM. At higher concentrations (>20 mM), it acts as non-competitive inhibitor. We performed our simulations at concentrations at which IMPase would only have residual activity hydrolyzing IP. However, inhibition by high concentrations is thought to be due to product trapping^31^,^32^,^35^. In a recent crystallographic study, it was concluded that both Li^+^ and Mg^2+^ do not interfere with the catalytic reaction, but stabilize the post-catalytic complex instead^31^. These data taken together, suggest that Mg-III can bind to IMPase to form a ternary complex even in the absence of inorganic phosphate, natural substrates or inhibitors, but we cannot estimate the affinity due to the millisecond binding timescale.

### Substrate pathway reconstruction

IMPase’s catalytic mechanism on IP’s hydrolysis has been the subject of several studies. For many years, the enzyme was thought to operate via two Mg^2+^ ions. Pollack *et al*.^16^ proposed a mechanism in which Mg-I acted as the water nucleophile activator while Mg-II as a stabilizer for the leaving inositol. More recent observations have favored a hydrolysis operating via three Mg^2+^ ions instead^20^. Despite the pre-catalytic complex being well characterized, the steps leading to its formation are not yet clear. Whereas some studies supported a random mechanism^22^, others inclined towards an ordered mechanism, with the substrate binding IMPase, and only Mg-III binding after^17^. Some other studies favored the presence of three Mg^2+^ in the absence of substrates^14^.

Assuming that once each of the Mg^2+^ ions binds in their corresponding positions and remain stable for timescales much longer than the substrate binding, we can consider bound ions as virtually covalent. For simplicity, we will refer to the protein states in which there are two and three Mg^2+^ bound per subunit as IMPase-II and IMPase-III. The exchange between IMPase-II and IMPase-III occurs in very long timescales, up to several milliseconds. This leads to a partition of IMPase’s conformational space into two different kinetic pathways. The binding of the natural substrate could present different relative affinities for IMPase-II and IMPase-III, leading to conformational selection^36^, and could be able to shift the equilibrium towards any of the conformers by induced-fit^37^.

In order to understand the sequence of events prior to the formation of the pre-catalytic complex, the binding was performed against these two protein states. The first system contained IMPase-II, and the two remaining Mg^2+^ corresponding to site III were randomly placed in solution. The second system contained IMPase-III, no Mg^2+^ ions present in bulk. A total of five IP molecules were placed around the enzyme in both cases. The final substrate and ion concentration were set to 12 and 5 mM, respectively. The two systems were therefore thermodynamically identical.

Taking into account the two systems, 155.8 μs of total sampling time was produced. The IP molecules carry two negative charges in the phosphate group, and therefore the interaction with other ligand molecules is avoided by electrostatic repulsion. Still, the ligands performed short-lived interactions among themselves or through Mg^2+^ bridges, the same way it could be expected in an experiment at this concentration. For our analysis, we treated each ligand interaction against IMPase as an independent trajectory from other ligands. The results show many IP binding events to IMPase-III and to IMPase-II, in coordination with Mg^2+^ or alone. In order to understand the main pathways of binding and provide kinetic estimates, an MSM was produced gathering the two simulation sets. In this analysis, the contacts between substrate and protein were mapped and geometrically clustered. After, each cluster was further split taking into account whether the Mg^2+^ ion was coordinated or not with IP’s phosphate group at a shorter distance than 4 Å (see Methods).

**Figure 4:**
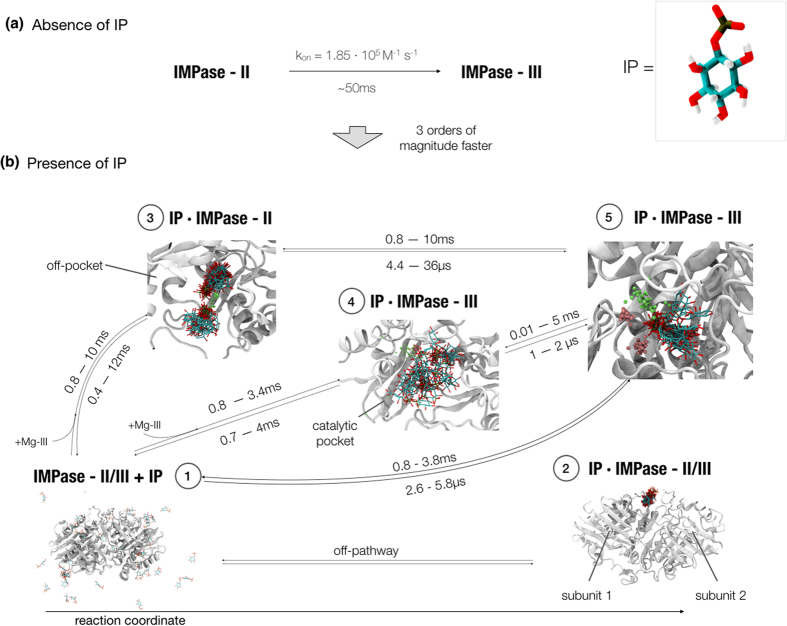
Overview of substrate mechanism of binding. **(a)** In the absence of substrate (IP), the conversion between the two IMPase conformers takes several milliseconds. **(b)** In the presence of substrate, the latter binds with very fast kinetics in a one-step mechanism to IMPase-III (state 5). The substrate is also able to bind IMPase-II in a two-step reaction by two different pathways. In the first case, it binds in a complex with Mg-III in the low millisecond timescale (state 4). It quickly rearranges to the crystal pose. In the second case, it binds to IMPase-II (state 3) in a pose distant from the Mg^2+^ dyad. It then rapidly acquires one free Mg-III and converts to the crystal pose.

**Figure 5:**
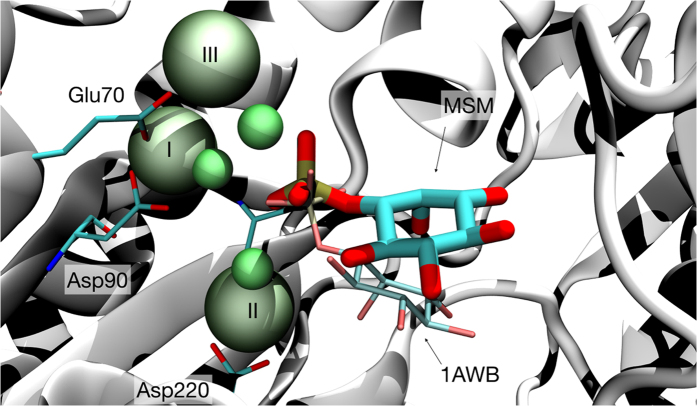
Pose from the most probable state in the analysis of IP binding superimposing with the available crystal structures (PDB code 1AWB) 20. Shown in green, the three ions, Ca^2+^ for the crystal structure (shown in a thinner representation), Mg^2+^ in our simulations (bulkier with labels on them). In cyan, the substrate IP, and in white the protein as obtained from simulations (MSM). Superimposed in a thinner representation using the same coloring method the substrate coordinates in 1AWB pdb. The protein residues coordinating the ions correspond to the simulations. The RMSD for this pose, taking into account the substrate heavy atoms, was 4.5 Å. (Fig. 4b, state 5).

Five final states showing IP free in solution, bound, or in metastable states were obtained. Figure 4b summarizes the transitions among them and their specific binding modes. State 1, corresponds to bulk or the initial state in the reaction pathway, that is to say, when IP is free in solution. State 2, located at the interface between subunits, does not directly convert to the other states without reverting to bulk. This binding pose is independent of IMPase’s coordination and occurs both in IMPase-II and III. IP does not interact to any Mg^2+^ ion in this pose. States 3 and 4, correspond to short-lived states in the pathway of IP binding. Lastly, state 5, corresponds to the bound pose in the catalytic pocket of IMPase-III. The pose obtained through our analysis, overlaps well with the crystal structure (Fig. 5). Under the case of a conventional non-covalent reversible binding, IP would bind with a Gibbs free energy of −7.1 ± 0.3 kcal/mol, as computed as the ratio between its off and on rates. Once bound, state 5 presents a residence time of several milliseconds. IMPase’s k_cat_ is 22 + 3 s^−1^ at 0.5 mM IP concentration, and therefore hydrolyzes an IP molecule in 45 ms. With this turnover number, IMPase-III plausibly hydrolyzes IP molecules once they have reached state 5, shifting metastable states 3 and 4 towards the bound pose. Full kinetic and quantitative data is presented in Table S1.

IP can reach the bound state through three different pathways of binding. The fastest binding route corresponds to the direct binding to IMPase-III from bulk. The average binding occurs in a time between 2.6 and 5.8 μs. A second, slower binding pathway consists on a two-step binding mechanism. The first rate-limiting step comprises the binding of the IP-Mg-III complex to IMPase-II’s catalytic pocket, occurring in 0.8–3.4 ms (state 4). Visual inspection of the trajectories leading to this state showed that although IP-Mg-III reaches the pocket as a complex, once inside the protein residues are able to dissociate the complex more than 4 Å apart. Thus, the MSM analysis detected a state represented by Mg-III and IP in disordered positions inside IMPase-II’s pocket. The formation of this metastable state is followed by a quick reordering of the complex towards the bound pose in 1–2 μs. The third, slowest mechanism consists on the binding IP alone to the vicinities of the catalytic pocket (state 3), with a time about 1–10 ms, followed by a much faster step occurring in a few microseconds. The longer rearrangement times for this third pathway regarding the second could be due to the formation of di-IP-Mg^2+^ complexes or the longer distance to the active site.

Looking at the different binding pathways in relative terms, we see that the first, single-step binding pathway occurs three orders of magnitude faster than the two others, and could in practical terms be the only pathway of binding. Assuming equal populations of the two IMPase forms, the substrate could reach the pre-hydrolysis pose over one thousand times through the direct pathway in the time it would need through the second or third pathways. Note, however, that the real populations of IMPase-II and IMPase-III remain unknown. We never observed the Mg-III’s unbinding event and neither the off-rate nor the equilibrium constant can be estimated or are present in literature.

From the binding event, we have estimated that the shift from IMPase-II to IMPase-III takes several milliseconds in the absence of IP, as deduced from the process’ on-rate of 1.85 · 10^5^ M^−1^ s^−1^ (Fig. 4a). However, in the presence of IP, the equilibrium is shifted to the right: once the substrate is bound to IMPase-II, the cofactor reaches site III in a few microseconds. These facts can easily be explained in terms of electrostatic repulsion. *apo*-IMPase presents a highly polar pocket composed by four acidic residues (Glu70, Asp90, Asp93, and Asp220), totaling up to four negative charges in close vicinity. The binding of Mg-I and Mg-II, each carrying two positive charges, neutralizes the pocket. The binding of Mg-III under these conditions would in principle not be very favorable, thus explaining its very slow kinetics. However, the natural substrate presents two negative charges on its phosphate group. The great differences we observe for substrate binding to IMPase-II and IMPase-III are also possibly a consequence of the pocket’s total charge differences: whereas IP’s binding to the doubly positively charged IMPase-III’s pocket is diffusion-controlled, the binding to neutral IMPase-II’s pocket takes a few milliseconds regardless its pathway.

## Discussion

We have fully characterized substrate and cofactor binding prior to the catalytic event. The first study concluded that the protein is able to form a ternary complex with Mg^2+^ ions, even in the absence of substrate, inhibitors or inorganic phosphate, but its population could not be reliably measured. Our study shows that Mg-I and II’s pocket identification is diffusion limited, whereas subsequent rearrangement of coordinating residues takes several milliseconds. The binding of Mg-III, although difficult to observe at physiological concentrations, could be recorded at 20 mM, giving a mean first passage time (mfpt) estimation of around 50 ms at 1 mM.

We have also provided an atomic-level description of substrate and cofactor cooperation and binding. IP is able to bind both IMPase-II and III forms to different extents. The substrate shows a very fast binding, occurring in a mean time of 4 μs, to IMPase-III. Additionally, the molecule is also able to bind to IMPase-II although in a slower fashion. Both either accompanied by Mg-III or alone it identifies IMPase-II in a millisecond two-step reaction. Interestingly, although event III is very difficult to observe even in high-throughput simulations as done here, the process speeds up by 3 orders of magnitude in the presence of IP. These facts are easily supported by the drastic net charge changes at IMPase’s pocket.

The mechanism presented complements previous studies on deciphering the order of substrate and cofactor binding. The most recent proposed studies agreed with the Leech *et al*.’s ordered mechanism, with substrate binding first and modifying or creating the binding site for one or two metals binding after^38^. However, this mechanism did not account with the 3-metal structures that later emerged^39^; and subsequent analysis proposed cofactor binding before and after substrate binding^17^,^32^. Our mechanism shows that substrate binding after cofactor is kinetically favored, however we have shown that cofactor binding before and along with substrate is also possible, and could be the only pathway in the scenario where IMPase-III’s population was marginal. Actually, the versatility here presented could explain the diversity in previous studies, and those which observed a random-ordered mechanism^35^,^40^. Biochemical experimental studies on IMPase’s gained a lot of attention two decades ago, however, difficulties to design bioavailable inhibitors prompted research on IMPase’s to come to a halt. We hope that future experiments can further progress our understanding of IMPase’s function and in particular help characterize the IMPase conformational space. We have used arguably the most advanced computational methods and infrastructure for exploring enzyme dynamics and whilst we are limited by the use of empirical force fields and possible difficulties of parameterization, we have been able to shed light onto important biochemical questions arising from previous experimental work. We note, however, that the picture of binding presented here, although capturing the different substrate binding routes, is only a portion of the complete conformational space. In particular, IMPase is known to present two segments in the entrance of its catalytic pocket (the β-hairpin region comprised by residues 30–40 and the short helix comprised by residues 70–75) which appear to be disordered in the absence of ions, and could undergo several rearrangements upon substrate and metal binding^17^,^39^. We have, of course, observed protein plasticity along all the simulation sets performed in these regions. Nevertheless, given all our structures started from active conformation such movements are not representative of the global protein conformation space. Helix and β-sheets formation occur at timescales much slower^41^ than our ensembles and we do not have enough data at this stage to provide a solid study on the role such segments.

In summary, we have used large-scale HTMD and been able to recapitulate the binding events of Mg ions and natural substrate at IMPase, and we identified structures close to the X-ray solutions. In addition, our methodology also provides important information about the competition, cooperativity and kinetics of the binding pathways in this complex three-body process. We propose that the pathway diversity seen here might not be a particular case of IMPase, but a general principle for ligand binding. The ligand-binding paradigm is rarely a two-body problem (drug and receptor) because, water, or in particular ions, can play a critical role. We note that the quantitative study of IMPase’s mode of action is a particularly challenging case. The highly polar nature of the enzyme’s pocket, the metal parameterization, long timescales of the processes and the three-body mechanism of binding might not be the case of other targets. Still, with the advances in computational infrastructure, forcefield and analysis methods we believe that this approach can provide insight to understanding binding pathways for difficult targets like this. We expect that in the near future approaches similar to the one presented will become common in the early stage of the drug discovery pipeline. Such a study can provide a deeper understanding of the binding processes and the endogenous population of the active site, essential aspects of lead finding and optimization.

## Material and Methods

### Simulation system setup and simulation parameters

Input coordinates for human IMPase protein were based on the PDB code 4AS4^13^. The AMBER FF12SB^42^ forcefield was used to describe all the protein parameters. Mg^2+^ parameters were taken from a previous study^43^ where the parameters were fitted against experimental data in order to provide a better description of their kinetic properties in water, also improving the phosphate binding description. All chemical entities were protonated with the OpenBabel software at pH 7.4^44^ and parameterized by the Antechamber 12 tool^45^. All the complexes were explicitly solvated by the LEAP module of the AMBER 12 software package in a TIP3P^46^ cubic water box with at least 12 Å distance around the complex and then electrically neutralized using K^+^ and Cl^−^ ions. The final size of the systems was about 90000 atoms. The different cofactor and substrate concentrations are specified in Table 1.

Each system was minimized and relaxed under NPT conditions for 1 ns at 1atm and 298 K using a time-step of 4 fs, rigid bonds, a cut-off of 9 Å and PME for long-range electrostatics. Heavy protein and ligand atoms were constrained by a 1 kcal/mol/Å^2^ spring constant during the equilibrations and gradually reduced. Production simulations were run using ACEMD over GPUGRID^47^ in the NVT ensemble using a Langevin thermostat with damping of 0.1 ps^−1^ and hydrogen mass repartitioning scheme to achieve timesteps of 4 fs^48^. The total simulation times are summarized in Table 1.

### Markov State Models

A Markov state model (MSM) for each of the systems was built from the molecular simulation trajectories. MSMs have been successfully used to reconstruct the equilibrium and kinetic properties in a large number of molecular systems^24^,^49^,^50^. By determining the frequency of transitions between conformational states we were able to construct a master equation which describes the dynamics between a set of conformational states. Relevant states are determined geometrically by clustering the simulation data onto a metric space (e.g. contact maps). In this case, a discrete description of the process was obtained by means of protein-ligand contact maps. The carbon alpha atoms in the protein and the Mg^2+^ atom or the heavy atoms for the substrate molecule were selected for the construction of contact maps along all the trajectories. Two atoms are in contact if their distance is less than 8 Å.

One of the most important requirements for constructing Markov models is to be able to finely discretize the slowest order parameters. TICA^51^ (time-lagged independent component analysis) is a method that projects the data on the slow order parameters, thus producing a very good discretization. After projecting the high-dimensional protein-ligand contact maps onto the three slowest processes found by TICA with a 2 ns lag-time, the n-dimensional projected data was clustered using the k-centers algorithm. 3 and 5-dimensional projections were used for the analysis of Mg^2+^ binding and pathway reconstruction. The master equation is then built as


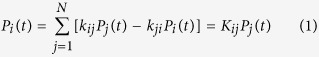


where P_i_(t) is the probability of state i at time t, and k_ij_ are the transition rates from j to i, and **K** = (K_ij_) is the rate matrix with elements K_ij_ = k_ij_ for i ≠ j and 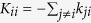. The master equation d**P**/dt = **K P** has solution with initial condition P(0) given by **P**(t) = **T**(t) **P**(0), where we defined the transition probability matrix T_ij_(t) = (exp[**K**t])_ij_ = p(i,t|j,0), i.e. the probability of being in state i at time t, given that the system was in state j at time 0. In practical terms, p_ij_(Δ*t*) is estimated from the simulation trajectories for a given lag time Δ*t* using a maximum likelihood estimator compatible with detailed balance^52^. The eigenvector **π** with eigenvalue 1 of the matrix *T*(Δ*t*) corresponds to the stationary, equilibrium probability. Higher eigenvectors correspond to exponentially decaying relaxation modes for which the relaxation timescale is computed by the eigenvalue as 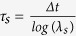, where *λ*_*s*_ is to the largest eigenvalue above 1. For long enough lag times the model will be Markovian, however every process faster than Δ*t* is lost. Therefore, the shortest lag is chosen for which the relaxation timescales do not show a dependence on the lag time Δ*t* anymore. In our case, we chose different lag times depending on the system, provided a good compromise between convergence in the implied timescale while being short enough to allow for sufficient statistical variance. Implied timescales and chosen lag times are shown in Table S2. Furthermore, although this fine discretization provides very good Markov models, it is necessary to reduce the amount of states to obtain a humanly interpretable model of the system in question. Therefore, the initial microstates can be lumped together into macrostates using kinetic information from the MSM eigenvector structure. Mean first passage times and commitor probabilities can also be calculated to obtain the relevant kinetics of the system^53^. Hence, the produced clusters were then lumped together into macrostates using the PCCA algorithm, each consisting of a set of kinetically similar clusters. For the specific case of substrate pathway binding, the analysis was performed as follows. The 3870 trajectories were split into five independent IP trajectories, as each simulation comprised five molecules. This set contained 7801920 frames, each of which was transformed into a ligand-protein alpha carbon contact map where two atoms were considered in contact when closer than 8 Å. After performing a TICA projection onto the 5 slowest order parameters, the data was geometrically clustered using the k-centers algorithm into 925 clusters. Each of these clusters was further split taking into account if the substrate’s phosphate group was in contact with any of the two Mg-III ions or not. 26 new clusters were created giving a total of 951 clusters, which were subsequently used in the MSM model. The microstates were finally combined into 5 macrostates by PCCA, and their transitions and binding modes are represented in Fig. 4b and Table S1. Only bulk, state 4 and 5 contained clusters in which Mg^2+^ and IP were in contact.

Errors were estimated for all properties using a bootstrapping technique. We performed 7 independent runs in which 20% of the trajectories were randomly eliminated and a new MSM was built after re-clustering. On each of these runs, the same parameters as described above were applied.

## Additional Information

**How to cite this article**: Ferruz, N. *et al*. Multibody cofactor and substrate molecular recognition in the myo-inositol monophosphatase enzyme. *Sci. Rep.* **6**, 30275; doi: 10.1038/srep30275 (2016).

## Supplementary Material

Supplementary Information

## Figures and Tables

**Figure 1:**
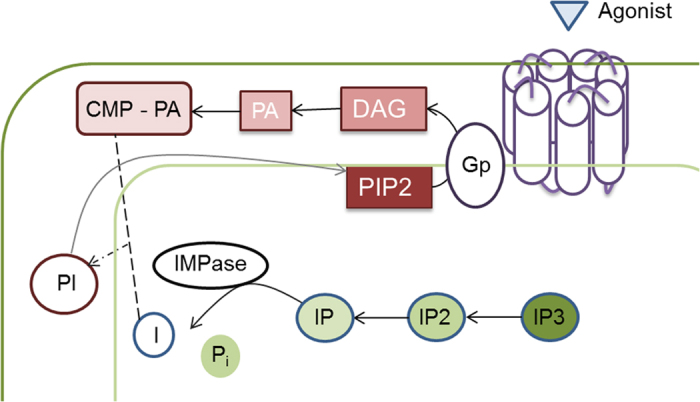
phosphatidyl inositol (PI) pathway and the role of IMPase. Phospholipase C, activated by G-protein-coupled receptors, hydrolyzes phosphatidylinositol 4,5 biphosphate (PIP2) to produce the two secondary messengers diacylglycerol (DAG) and inositol 1,4,5-triphosphate (IP3), which activate cellular responses. DAG is phosphorylated to give phosphatidic acid (PA) and then ligated to a cytidine giving cytidine monophosphorylphosphatidate (CMP-PA). The latter can be attached to a *myo*-inositol molecule and form phosphatidylinositol (PI) which can be then converted to PIP2. IP3 then undergoes a series of hydrolysis to generate inositol monophosphates, from which the final hydrolysis of *myo*-inositol monophosphate (IP) to inositol and inorganic phosphate (Pi) is catalyzed by myo-inositol monophosphatase (IMPase)^40^. Released free inositol (I) is then recycled into PIP2. IMPase is also involved in the *de novo* synthesis of inositol by converting glucose-6-phosphate into IP.

**Figure 2:**
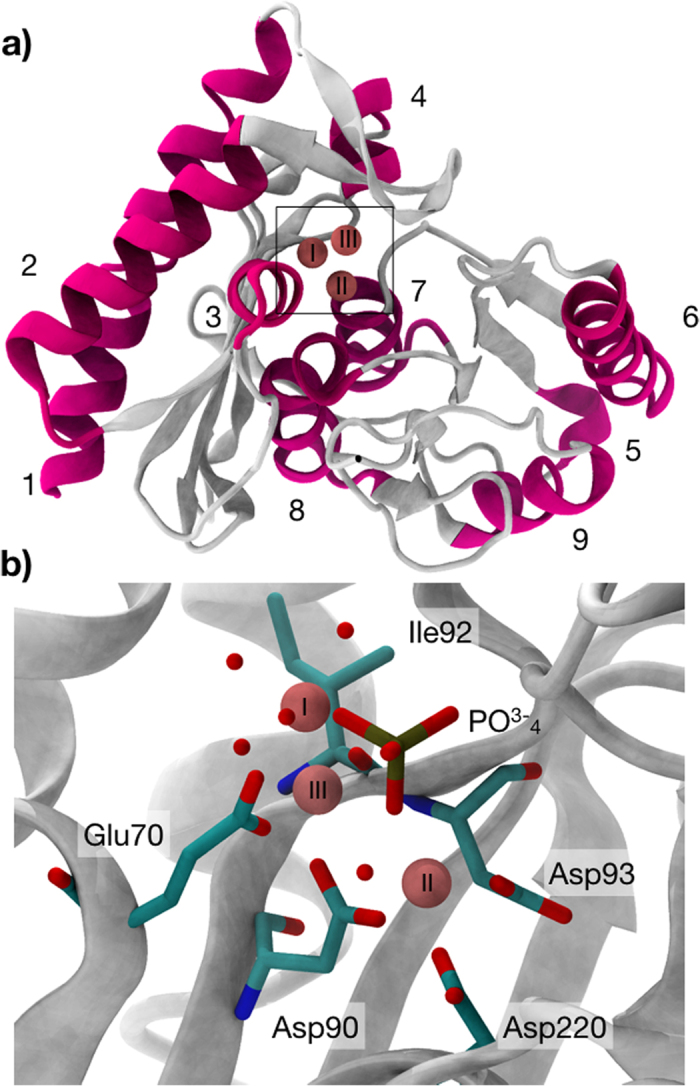
Structural features IMPase. PDB code 4AS4. **(a)** Cartoon diagram of the overall folding of one IMPase monomer depicting the penta-layered αβαβα sandwich. Alpha helices are coloured in pink whereas the two sets of beta sheets are shown in white. Mg^2+^ ions are shown as spheres. (**b**) The catalytic site showing the three Mg^2+^ ions. Mg-I is coordinated with Glu70, Asp90, and Ile92 and three water molecules, Mg-II with Asp90, Asp93 and Asp220 and three water molecules, one of them shared with Mg-I. Low-affinity Mg-III interacts with Glu70 and five water molecules.

**Table 1:** Summary of performed simulations, grouped by batches.

	**Simulation time (μs)**	**No. Mg**^**2+**^ **bound per monomer**^a^	**No. Mg**^**2+**^ **free in bulk**^b^	**Ligand of study**	**Ligand concentration (mM)**
**1 (event I)**	183	0	1	Mg^2+^	3
**2 (event II)**	93	1	1	Mg^2+^	8
**3 (event III)**	236	2	1	Mg^2+^	13
**4 (event III)**	100	2	16	Mg^2+^	54
**5 (IP-2MG)**	407 (81.4)	2	2	IP	12
**6 (IP-3MG)**	372 (74.4)	3	0 (K^+^)	IP	12

For each group, the number of bound Mg^2+^ ions or free in solution Mg^2+^, the ligand object of study by MSM is specified and its concentration is specified. In the case of the substrate analysis (batches 5 and 6), 407 and 372 μs were analyzed, respectively, as the simulations contained 5 ligands per box that were treated independently. The actual simulation time produced per batch is shown in parenthesis.

^a^Number of Mg^2+^ that were placed on the catalytic sites on each monomer at the beginning of the simulation.

^b^Number of Mg^2+^ that were placed in bulk at the beginning of the simulation.

**Table 2:** Kinetic and thermodynamic characterization of Mg^2+^ binding obtained by MSM analysis.

	**Monomer 1**	**Monomer 2**
**ΔG**^**0**^ **(kcal/mol)**	−3.7 ± 0.1	−3.8 ± 0.1
**k**_**on**_ **(M**^**−1**^ **s**^**−1**^)	6.4 · 10^8^ ± 1.2· 10^8^	4.8 · 10^8^ ± 2.2 · 10^7^
**k**_**off**_ **(s**^**−1**^)	1.3 · 10^6^ ± 2.2· 10^5^	7.5 · 10^5^ ± 7.6· 10^5^
