# Supplementary material for: Multibody cofactor and substrate molecular recognition in the myo-inositol monophosphatase enzyme
Source: Sci Rep. 2016 Jul 21;6:30275. doi: 10.1038/srep30275 (PMC4954947; doi:10.1038/srep30275)
Supplement: Supplementary Information [file srep30275-s1.pdf]

Supporting Information for:

Multibody cofactor and substrate molecular recognition in the *myo*-inositol monophosphatase enzyme

Noelia Ferruz,[†] Gary Tresadern,[§] Antonio Lucena-Pineda,[‡] and Gianni De Fabritiis, [†,¥,*]

[†]*Computational Biophysics Laboratory (GRIB-IMIM), Universitat Pompeu Fabra, Barcelona Biomedical Research Park (PRBB), Doctor Aiguader 88, 08003, Barcelona, Spain.*
[§] *Research Informatics, Janssen Research and Development, Janssen Cilag S A, Calle Jarama 75, Poligono Industrial, Toledo 45007, Spain*
[‡]*Centro de Investigación Príncipe Felipe, 46012 Valencia, Spain*
[¥]*Institució Catalana de Recerca i Estudis Avançats, Passeig Lluis Companys 23, 08010 Barcelona, Spain*

FIGURES

Figure S1: Binding mode of Mg-III is found at the interface between monomers, mainly interacting with Asp-209.

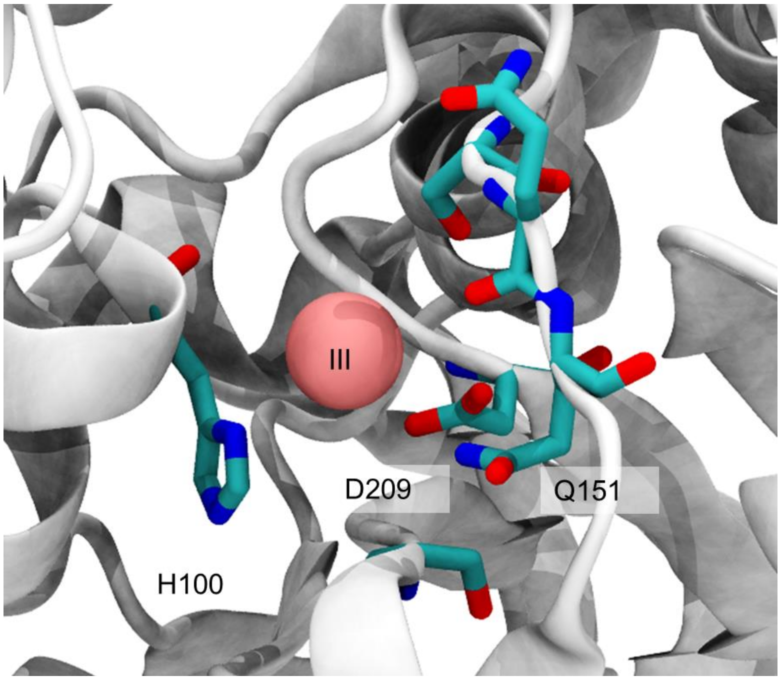

TABLES

Table S1: Full quantitative and kinetic data for the binding mechanism of substrate binding. The data is shown as a mean with its standard deviation, unless shown as an interval in the cases of low precision.

	**ΔG_{kin} (kcal/mol)**	**k_{on} ($s^{-1}\cdot M^{-1}$)**	**k_{off} (s^{-1})**
**State 1**	0	-	-
**State 2**	-	$985 – 5.2 \cdot 10^{4}$	$466 – 7.2 \cdot 10^{5}$
**State 3**	-2.3 ± 1.0	$3.7 \cdot 10^{4} – 4.9 \cdot 10^{5}$	702 ± 397
**State 4**	-5.5 ± 2.0	$7.5 \cdot 10^{4}$ -- $1.5 \cdot 10^{8}$	745 ± 388
**State 5**	-7.1 ± 0.3	$(-1.0 \pm 0.3) \cdot 10^{8}$	666 ± 350

Table S2: Timescales and lag times obtained for each of the three MSM analyses performed in this work. Simulation numbers correspond to the batches presented in Table 1 in the main manuscript. The error bars in the timescales are obtained after bootstrapping at the 80% of the original data for ten times, each of the replicas having a different clustering.

**Simulation batch**	**Implied Timescales**	**Lag time (ns)**
1	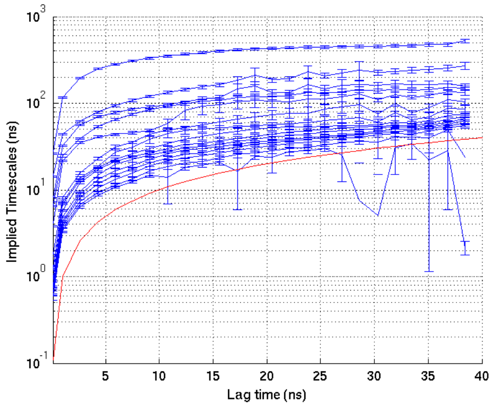	15

**2**	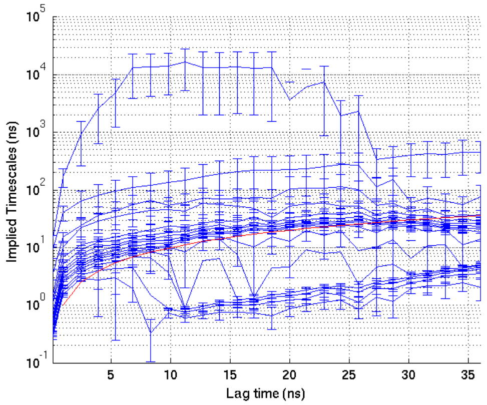Implied Timescales (ns) Lag time (ns)	15
**5-6**	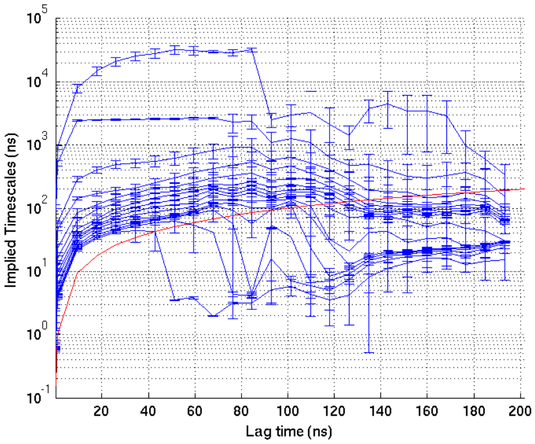Implied Timescales (ns) Lag time (ns)	50
